# *Contracaecum fioravantii* n. sp. from the black-crowned night heron (*Nycticorax nycticorax*)

**DOI:** 10.1017/S0031182026102558

**Published:** 2026-07

**Authors:** Perla Tedesco, Nadav Davidovich, Sean A. Locke, Andrea Gustinelli, Monica Caffara

**Affiliations:** 1 Department Veterinary Medical Sciences, University of Bologna, Ozzano Emilia, Italy; 2 Pathology and Diagnostics Division, Kimron Veterinary Institute, Israel; 3 Department of Biology, University of Puerto Rico, Puerto Rico

**Keywords:** *Contracaecum fioravantii* n. sp, integrative taxonomy, *Nycticorax nycticorax*, piscivorous birds

## Abstract

The genus *Contracaecum* comprises anisakid nematodes with complex life cycles involving aquatic intermediate hosts and piscivorous birds as definitive hosts. The black-crowned night heron (*Nycticorax nycticorax*) is a widespread ardeid species known to host diverse helminth communities, including *Contracaecum* spp., although species-level identification is often lacking. In this study, we describe a new species, *Contracaecum fioravantii* n. sp., based on an integrative morphological and molecular approach. Adult nematodes were collected from 3 *N. nycticorax* individuals in Israel and examined using light and scanning electron microscopy. The new species is characterized by a distinct combination of morphological features, including robust, subequal spicules, a sagittate tail tip in males, and a specific arrangement of caudal papillae and phasmids, distinguishing it from closely related taxa such as *C. microcephalum* and members of the *C. rudolphii* complex. Multivariate morphometric analyses supported its separation from congeners. Molecular characterization based on ITS (Internal Transcribed Spacers) rDNA and *cox2* mtDNA sequences revealed high similarity with previously unidentified *Contracaecum* spp. from ardeid hosts in China, and phylogenetic analyses placed the new species in a well-supported, distinct clade. These findings highlight the hidden diversity within *Contracaecum* and underscore the importance of combining morphological and molecular data for accurate species delimitation. The identification of *C. fioravantii* n. sp. contributes to a better understanding of host–parasite relationships in piscivorous birds and suggests a wider geographic distribution linked to migratory pathways of *N. nycticorax*.

## Graphical abstract

**Figure:**
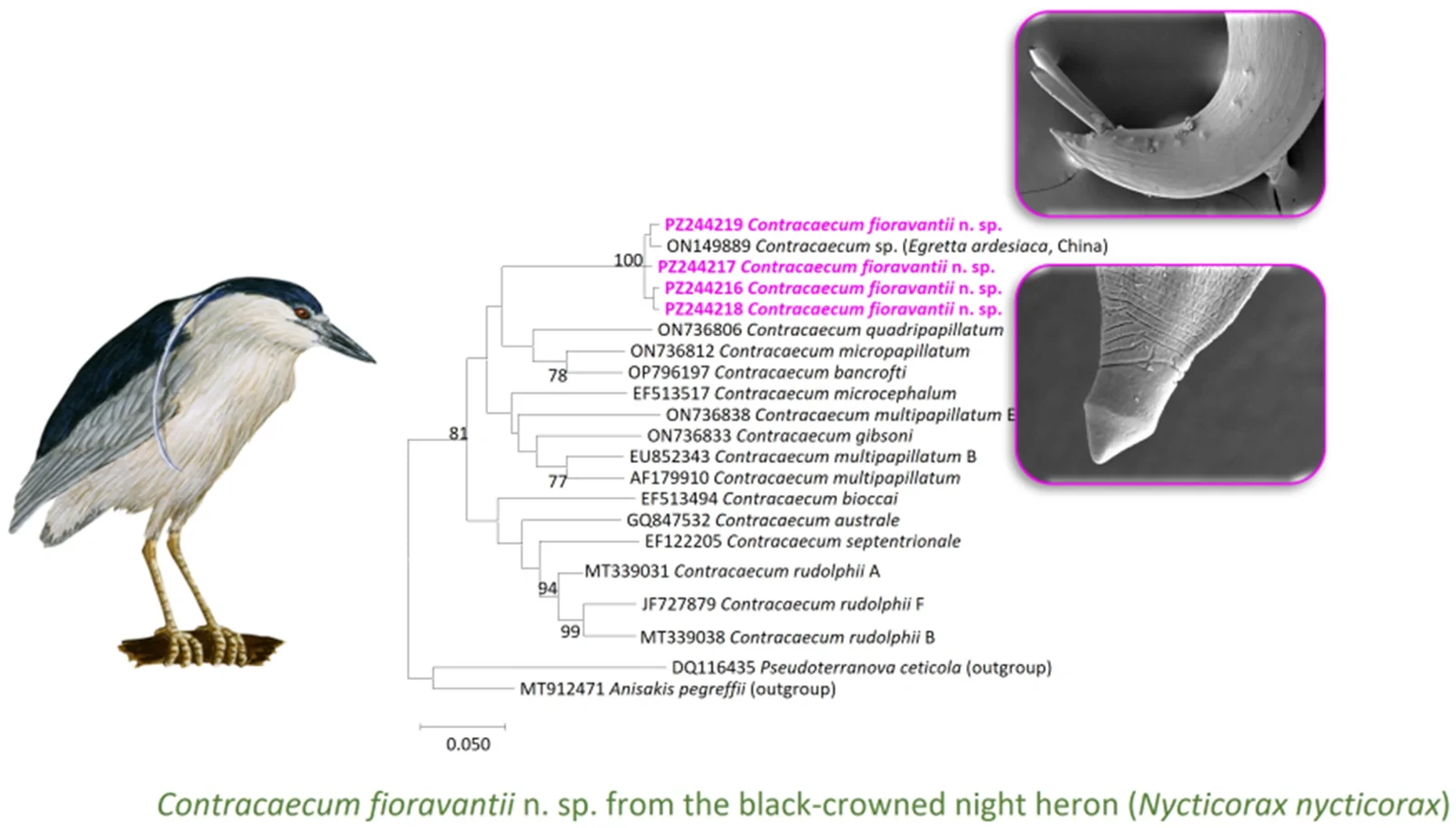

## Introduction

The genus *Contracaecum* Railliet and Henry 1912 comprises heteroxenous parasites with life cycles dependent on aquatic intermediate hosts (crustaceans, fish) and definitive hosts that are predominantly piscivorous birds and, less frequently, marine mammals (e.g. pinnipeds) (Abollo et al., 2001; Mattiucci et al., 2020). Humans (accidental hosts) can become infected by ingesting raw or undercooked fish carrying third-stage larvae (Shamsi and Butcher, 2011).

In freshwater systems, herons (Ardeidae) contribute substantially to the transmission and maintenance of *Contracaecum* spp. (Barson and Marshall, 2004; Deng et al., 2022). For example, 6 nematode species, including *Contracaecum*, were found in 6 ardeids in Spain (Navarro et al., 2005). In a breeding colony in central Poland (Dziekońska-rynko et al., 2015), larval and adult stages of *C. rudolphii* Hartwich 1964 were found in all grey heron (*Ardea cinerea* L.) chicks examined, demonstrating that infections with this parasite occur very early in life, with possible adverse impacts on host health, and indicating a transfer of parasites from adult birds to chicks during feeding.

The black-crowned night heron (*Nycticorax nycticorax* L.), an ardeid host of *Contracaecum* spp. with a wide geographical range, has a broad breeding range across Eurasia, extending from southern and central Europe eastward through western and central Asia into China and Japan. In Europe, breeding populations exist from Iberia through Greece and Turkey, reaching northward into France, the Netherlands and Russia; northern European populations are largely migratory, wintering in sub-Saharan Africa. In Asia, *N. nycticorax* breeds from the Caspian region eastward into China and Japan; many northern populations migrate southward, while southern Asian populations tend to be resident year-round (IUCN, 2025).

Colonies at Israel’s Huleh Nature Reserve include both resident and migratory individuals, highlighting partial migration behaviours in the Middle East (Ashkenazi and Yom-tov, 1997). In eastern Spain, characterized by mild temperatures and high food availability, winter breeding occurs as late as February (Dies et al., 2003).

During the breeding season in Eurasia, *N. nycticorax* exhibits an opportunistic feeding behaviour, with fish as the dominant prey across populations. In northern Honshū (Japan), black-crowned night herons primarily consumed Japanese loach (*Misgurnus anguillicaudatus* Cantor 1842), shifting foraging sites between rivers during daylight and irrigated paddy fields at night to match prey activity rhythms (Endo and Sawara, 2000). In western Spain, nestling diets comprised over 93% fish, predominantly *Barbus comiza* Steindachner 1864, while amphibians were almost absent, likely due to local river fauna dynamics (Pérez et al., 1991). In the floodplain wetlands of the Paraná River (Argentina), fish were also the most important dietary item of black-crowned night herons, followed by insects and crustaceans (Quiroga et al., 2013). Together, these studies show a strong piscivorous preference among widely separated populations of this species.

*Nycticorax nycticorax* harbours a diverse community of helminths, including nematodes of the genus *Contracaecum*, which are commonly reported across different geographic regions. European studies have shown a notable prevalence of *Contracaecum* species (e.g. *C. microcephalum* (Rudolphi 1819 (Baylis 1920)), suggesting that this heron plays an important role in the parasite’s life cycle, likely through trophic transmission associated with the consumption of infected fish (Sitko and Heneberg, 2015; Syrota et al., 2021).

Adult stages of *Contracaecum* have been recovered from *N. nycticorax* in southern Italy (Santoro et al., 2026), Japan (Yoshida et al., 2012), the USA (Groves et al., 2025), China (Zhang et al., 2021) and Iraq (Mhaisen and Abdul-ameer, 2021). Infection levels vary geographically and are influenced by local ecological factors such as diet and intermediate host availability. With respect to Israel and the Middle East, Ashkenazi and Yom-tov (1996) reported that black-crowned night herons in the Hula Valley, Israel, were found to harbour *C. microcephalum* larvae in their stomachs.

Although *N. nycticorax* represents an important host for *Contracaecum* in aquatic ecosystems, many reports in this host species do not identify the parasite to species level. A species-level understanding of *Contracaecum* diversity, distribution and pathogenic potential in ardeids is essential for elucidating host–parasite dynamics and assessing ecological and potential public health implications. In the present work, which is part of a larger study of parasites of piscivorous birds in Israel, morphological and molecular methods are employed to describe a new species of *Contracaecum* from *N. nycticorax* in Israel.

## Materials and methods

### Sampling and morphological study

Fourteen *Contracaecum* specimens were collected from the gastric mucosa of 3 black-crowned night herons (*N. nycticorax*) from a fish farm belong to Gesher, situated along the Jordan Rift Valley, Israel, collected under permits 2020/42659 and 2021/42855 from the Israel Nature and Parks Authority. The nematodes were taken from freshly processed hosts, washed in saline and preserved in 70% ethanol for morphological and molecular analysis.

A total of 7 male and 3 female adult worms were examined under a dissection microscope to assess gross morphological characteristics and record total length. Detailed morphological observations were subsequently conducted using a light microscope (Nikon Ci-L Plus, Nikon Europe B. V. Amstelveen, The Netherlands) equipped with a Nikon DS-Fi1 digital camera and image acquisition software (Nikon NIS-Elements U D-SUA). Except where noted, measurements are given in micrometres. The central region of each specimen, which lacks taxonomically diagnostic features, was excised for genomic DNA extraction. The anterior and posterior regions of the body were cleared in Amman’s lactophenol to facilitate visualization and measurement of internal structures. Morphometric analysis was carried out following Hartwich (1964) and Baruš et al. (1978), while the nomenclature of the caudal structures of males follows Fagerholm (1991).

For scanning electron microscopy (SEM), anterior and posterior portions of male and female specimens were dehydrated through a graded ethanol series, dried in hexamethyldisilazane, sputter-coated with gold palladium and observed using a Phenom XL G2 Desktop SEM (Thermo Fisher Scientific, Eindhoven, The Netherlands) operating at 5 kV.

The morphology of the specimens was quantitatively compared to those of other species using multivariate analysis implemented in Primer-E (Auckland, New Zealand). These analyses employed morphometrics of 10 specimens (7 male, 3 female) from the present study and up to 3 data points from other species, consisting of the minimum, maximum and mean reported in other studies. For each variable, data were normalized (the mean subtracted from each observed value, and the result divided by the standard deviation). Analysis of similarity (ANOSIM) with 9999 permutations was used to test for differences among species (crossed by worm sex) in the matrix of Euclidean distances among specimens. The resemblance matrix was visualized using a metric multidimensional scaling (MDS) ordination, after removing variables and species with >50% missing data.

### Molecular study

For molecular analysis, genomic DNA was extracted using a PureLink® Genomic DNA Kit (Life Technologies, Carlsbad, CA, USA) following the manufacturer’s instructions. The ITS rDNA was amplified with primers NC5_f (5′-GTAGGTGAACCTGCGGAAGGATCATT-3′) and NC2_r (5′-TTAGTTTCTTCCTCCGCT-3′) (Zhu et al., 1998), while a fragment of *cox*2 mtDNA was amplified from with primers 211_f (5′-TTTTCTAGTTATATAGATTGRTTTYAT-3′) and 210_r (5′-CACCAACTCTTAAAATTATC-3′) of Mattiucci et al. (2008) following the same protocol. The PCR products were electrophoresed on a 1% agarose gel stained with SYBR Safe DNA Gel Stain (Thermo Fisher Scientific, Carlsbad, CA, USA) in 0.5× TBE. For sequencing, the amplicons were excised and purified by Nucleo-Spin Gel and PCR Clean-up (Mackerey-Nagel, Düren, Germany), and sequenced with an ABI 3730 DNA analyser (StarSEQ, Mainz, Germany). The DNA trace files were assembled with Contig Express (VectorNTI Advance 11 software, Invitrogen, Carlsbad, CA, USA), and the consensus sequences of the ITS rDNA after separating the 2 regions (ITS1 and ITS2) and *cox*2 mtDNA were compared with published data by BLAST tools (Altschul et al., 1990). Multiple sequence alignments were performed using BioEdit 7.2.5 (Hall, 1999). Genetic distances (uncorrected *p*) and maximum-likelihood (ML) trees (using GTR + G + I substitution model for both ITS and *cox*2 and bootstrap of 1000 replicates) were obtained using MEGA12 (Kumar et al., 2024). The ITS1 and ITS2 rDNA sequences were concatenated and used to build an ML tree together with the sequences of *Contracaecum* spp. reported by Mattiucci et al. (2020) together with other *Contracaecum* sequences retrieved from GenBank. The *cox*2 gene was also aligned with the sequences reported by Mattiucci et al. (2020), with *Pseudoterranova ceticola* (DQ116435) and *Anisakis pegreffii* (MT912471) as outgroups. The sequences generated in this study have ${}^{\sim }$been deposited in GenBank under accession numbers PZ240692-69, PZ240700-01 (ITS rDNA) and PZ244216-19 (*cox2* mtDNA).

## Results

### Morphological description

**Figure 1.**
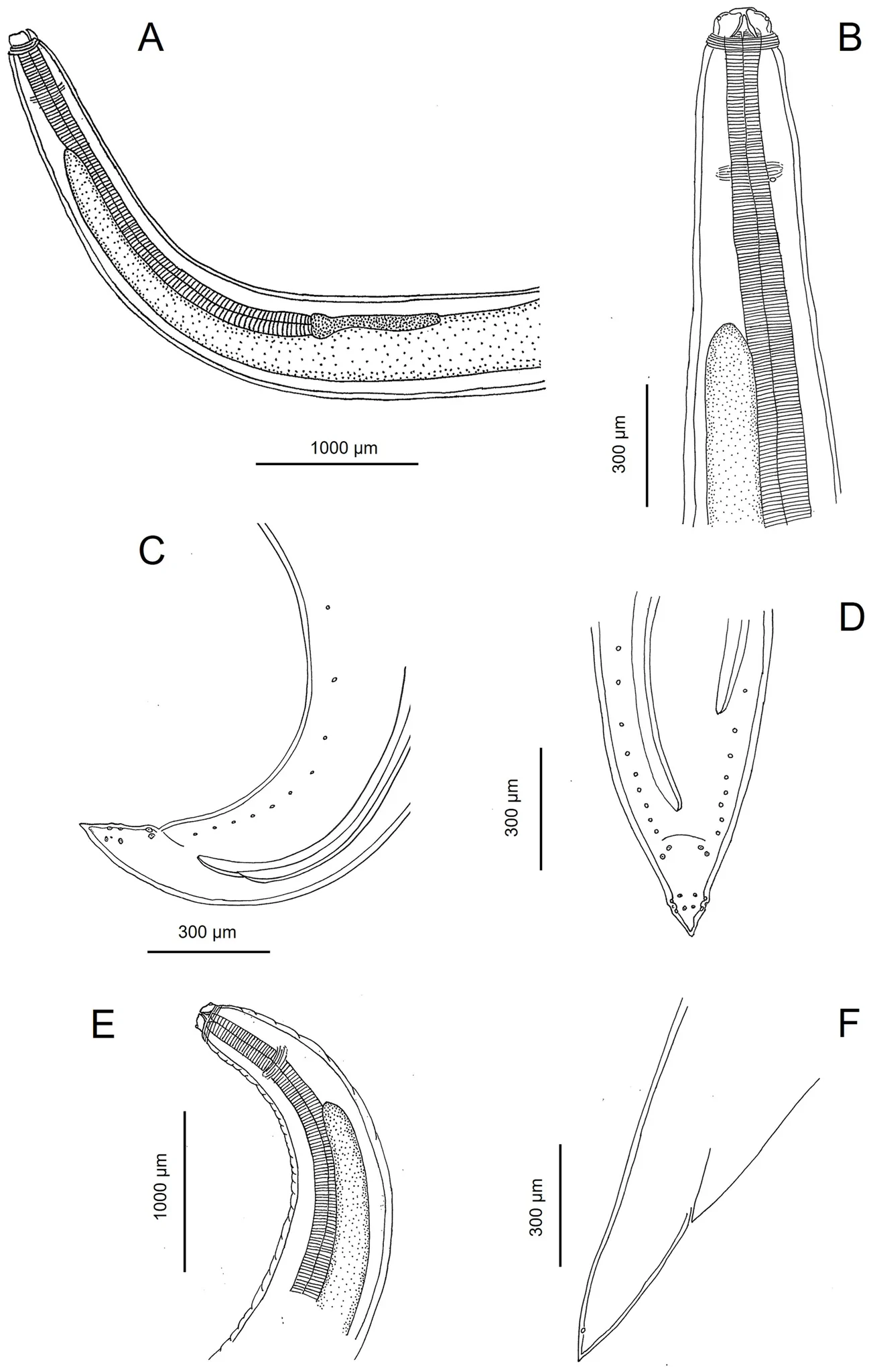
Line drawing of adult male and female specimens of *C. fioravantii* from *N. nycticorax*: (A) and (B) male specimen, anterior end; (C) and (D) male specimen, posterior end; (E) female specimen, anterior end; and (F) female specimen, posterior end.

**Figure 2.**
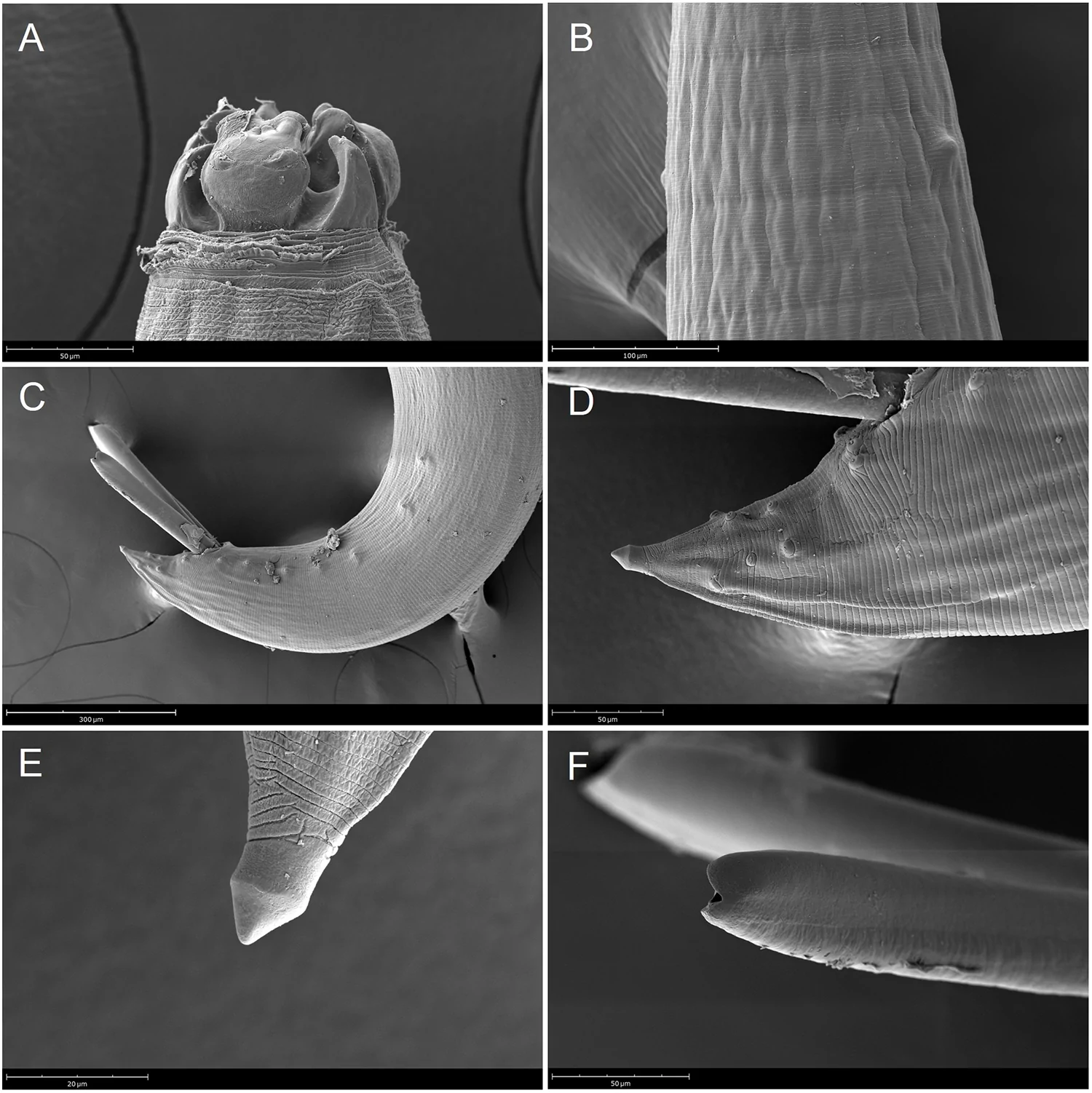
Adult male of *C. fioravantii* from *N. nycticorax*, SEM: (A) anterior end with detail of dorsal lip and interlabium (scale bar 50 µm); (B) detail of amphid (scale bar 100 µm); (C) posterior end with everted spicules (scale bar 300 µm); (D) detail of distal papillae (scale bar 50 µm); (E) detail of caudal tip (scale bar 20 µm); and (F) detail of spicule tip (scale bar 50 µm).

**Figure 3.**
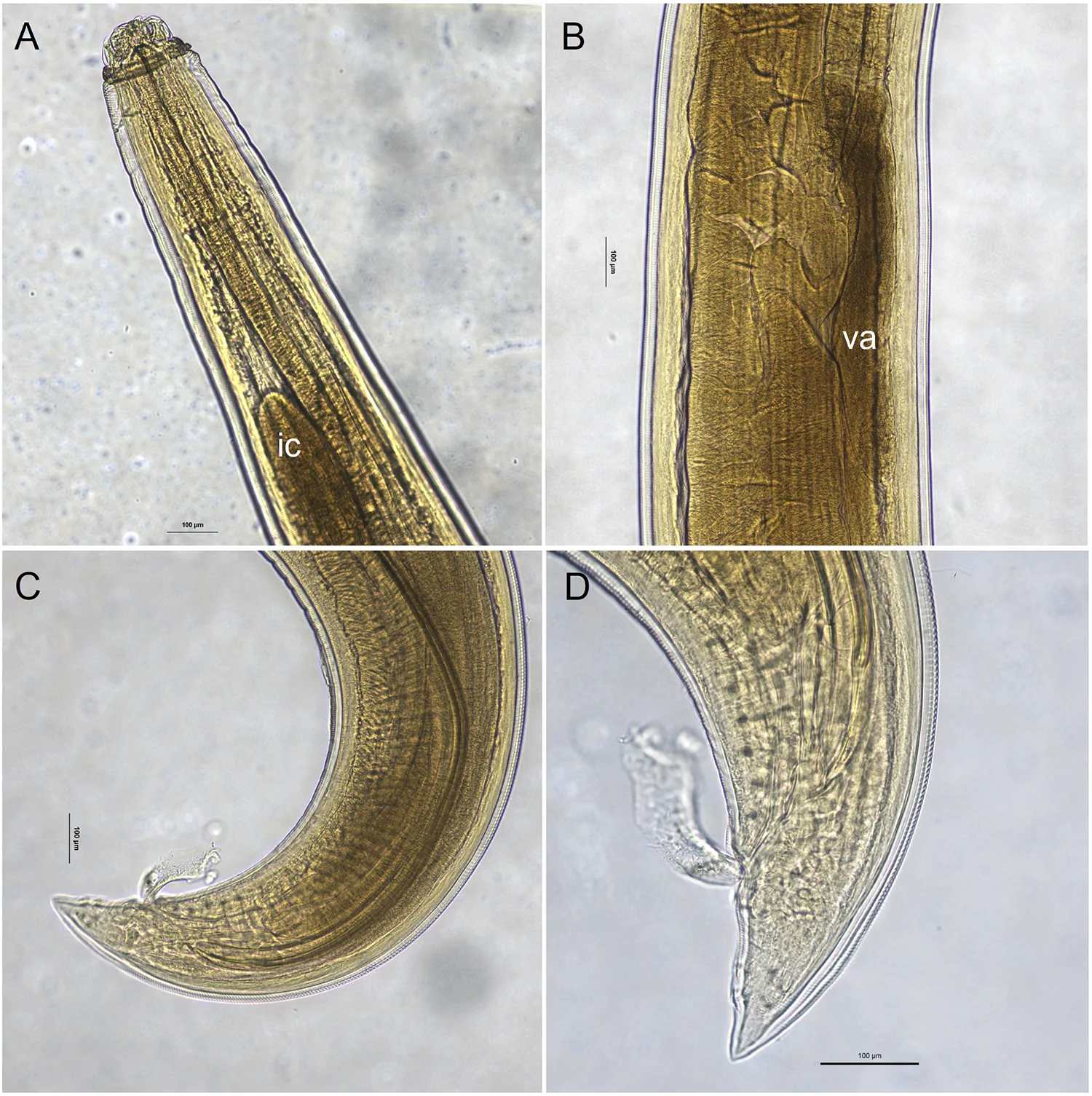
Adult male of *C. fioravantii* from *N. nycticorax*, light microscopy: (A) anterior end with terminal part of intestinal caecum (ic); (B) detail of ventricular appendix (va); (C) posterior end with spicules (sp); and (D) detail of posterior end with caudal papillae. All scale bars 100 µm.

**Figure 4.**
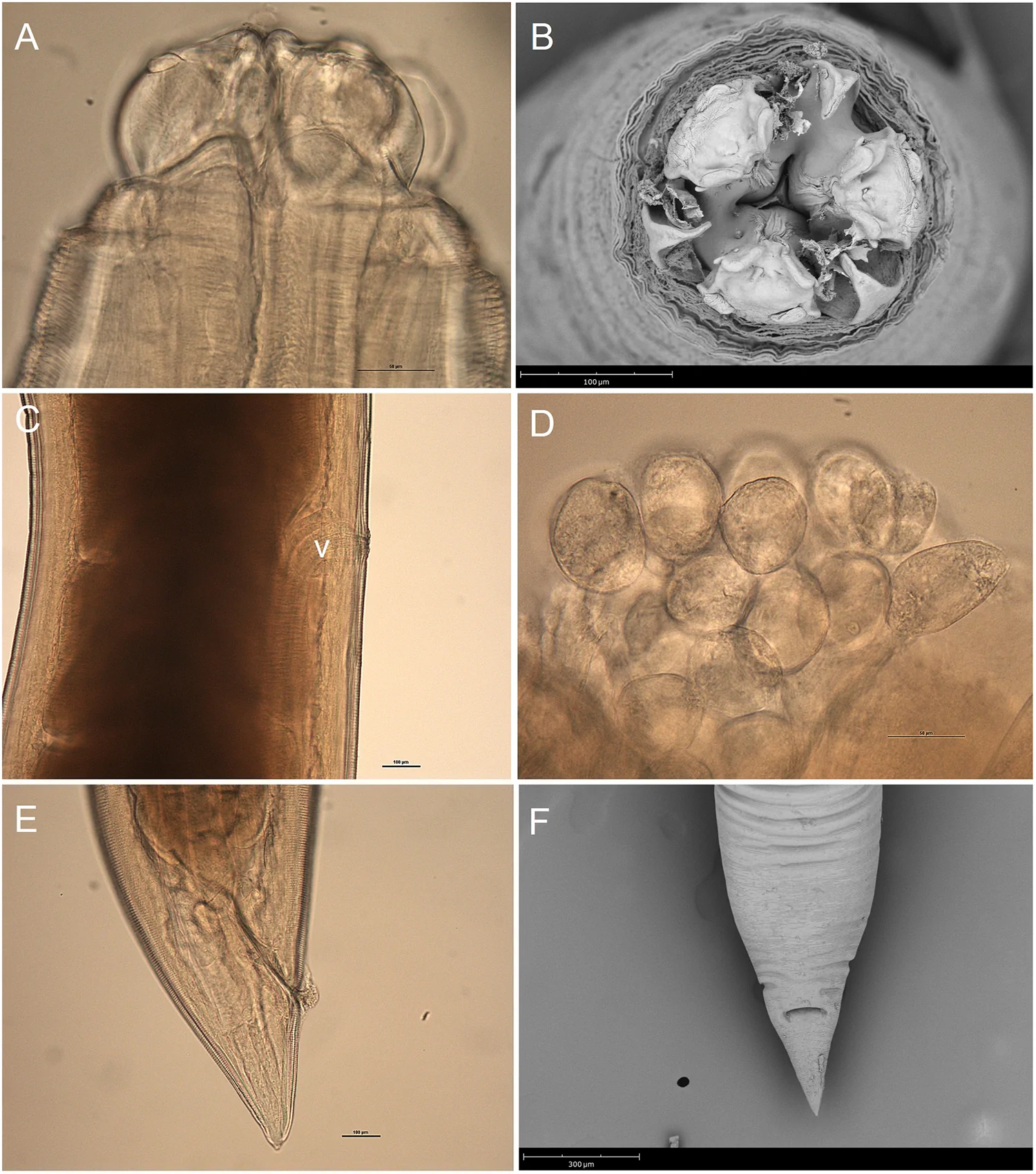
Adult female of *C. fioravantii* from *N. nycticorax*, light and SEM: (A) anterior end, lateral view (scale bar 50 µm); (B) anterior end, apical view (scale bar 100 µm); (C) detail of vulva (v) (scale bar 100 µm); (D) eggs (scale bar 50 µm); (E) posterior end, lateral view (scale bar 100 µm); and (F) posterior end, ventral view (scale bar 300 µm).

***Contracaecum fioravantii* n. sp. (*n* = 10) (**Figures 1**–**4)

Host: *N. nycticorax*

Site in host: stomach

Locality: Gesher, fish farm along the Jordan Rift Valley, Israel

Type-material: Holotype (1 male) and allotype (1 female) are deposited at the Helminthological Collection of the Institute of Parasitology, Biology Centre of the Czech Academy of Sciences, České Budějovice, Czech Republic (IPCAS N-1295).

Representative DNA sequences: PZ240692-69, PZ240700-01 (ITS rDNA) and PZ244216-19 (*cox2* mtDNA).

Etymology: *Contracaecum fioravantii* n. sp. is named after Professor Marialetizia Fioravanti, whose scientific contributions and passion for parasitology have inspired our work and fostered our appreciation of parasites.

Adult stage: Body stout. Cuticle transversally striated (Figures 1–4). Lips longer than wide (Figure 2A), 1 dorsal and 2 subventral (Figure 4B). Two oval cephalic papillae on dorsal lip and a single papilla on each subventral lip (Figures 2A, 4B). Interlabium reaching approximately 4/5 of lip length, wider at base, with pointed tip. Excretory pore at base of lips. Collar area short, with fine cuticular annulations interrupted laterally (Figure 2A). Amphids approximately at level of nerve ring (Figures 1B, 2B). Oesophagus muscular, ending in round ventriculus with short ventricular appendix (Figures 1A, 3B). Intestinal caecum 3 times longer than ventricular appendix (Figures 1A, 3A).

Males (*n* = 7). Body length 11–35 (18 ± 8.36) mm. Labia 55–98 (69 ± 17.45), interlabia 49–89 (60 ± 16.63). Oesophagus 2584–3905 (3099 ± 552.20). Nerve ring 325–638 (470 ± 102.61) from anterior extremity. Amphids 319–569 (431 ± 90.04) from anterior extremity. Intestinal caecum 1968–3306 (2479 ± 481.78) long. Ventricular appendix 845–1441 (1067 ± 215.70) long. Spicules subequal, robust, folded, with longitudinal alae (Figures 1C, D, 2F, 3C). Right spicule 2520–3849 (3247 ± 539.82), left spicule 2538–4043 (3309 ± 566.66). 21–23 pairs of simple proximal caudal papillae forming 2 subventral rows, 2 pairs of paracloacal papillae, 2 pairs of distal subventral papillae and 2 pairs of distal sublateral papillae, 1 pair of phasmids between distal subventral and distal sublateral papillae (Figures 1D, 2C, D). Tail 205–364 (270 ± 53.51), with sagittate tip (Figure 2E).

Females (*n* = 3). Body length 16–27 (22 ± 5.56) mm. Labia 89–143 (111 ± 28.54), interlabia 79–105 (91 ± 13.29). Oesophagus 4341–5368 (4948 ± 538.27) long. Nerve ring 497–676 (583 ± 90.08) from anterior extremity. Intestinal caecum 3455–4655 (4176 ± 636.08) long. Ventricular appendix 1194–1522 (1371 ± 165.42) long. Tail conical, 494–632 (544 ± 76.93) long, with pointed tip (Figures 4E, F). Vulva 8798–15274 (11623 ± 3316.48) from anterior extremity (Figure 4C). Eggs smooth, ovoid (Figure 4D), 125–171 (146 ± 171.1) long, 111–147 (128 ± 146.85) wide.

### Remarks

*Contracaecum fioravantii* n. sp. presents a few similarities but also marked morphometric differences with respect to other *Contracaecum* species previously reported in *N. nycticorax*. Among other, *C. microcephalum* shows a similar pattern of distal papillae and a similar appearance of the spicule tip, however the spicules in *C. microcephalum* are on average smaller than in *C. fioravantii* n. sp. (2600–3120 vs 3247–3309 right-left spicule, respectively); furthermore, the 2 species differ with respect to the appearance of the caudal tip, which is pointed in *C. microcephalum* (Cram, 1927; Baruš et al., 1978) and sagittate in *C. fioravantii* n. sp. The arrangement of distal papillae in *C. fioravantii* n. sp. is similar to that of members of the *C. rudolphii* complex. However, in *C. fioravantii* n. sp., phasmids are located between distal subventral and distal sublateral papillae, while in *C. rudolphii* they are dorsal to distal sublateral papillae. More importantly, spicule morphometry is markedly distinct, with considerably longer, narrower spicules in *C. rudolphii* (8054 right/7756 left) and shorter, more robust spicules in *C. fioravantii* n. sp. Furthermore, the appearance of spicule tip is markedly distinct (Figure 2F, as compared to SEM micrographs of *C. rudolphii* in Caffara et al., 2023b).

**Figure 5.**
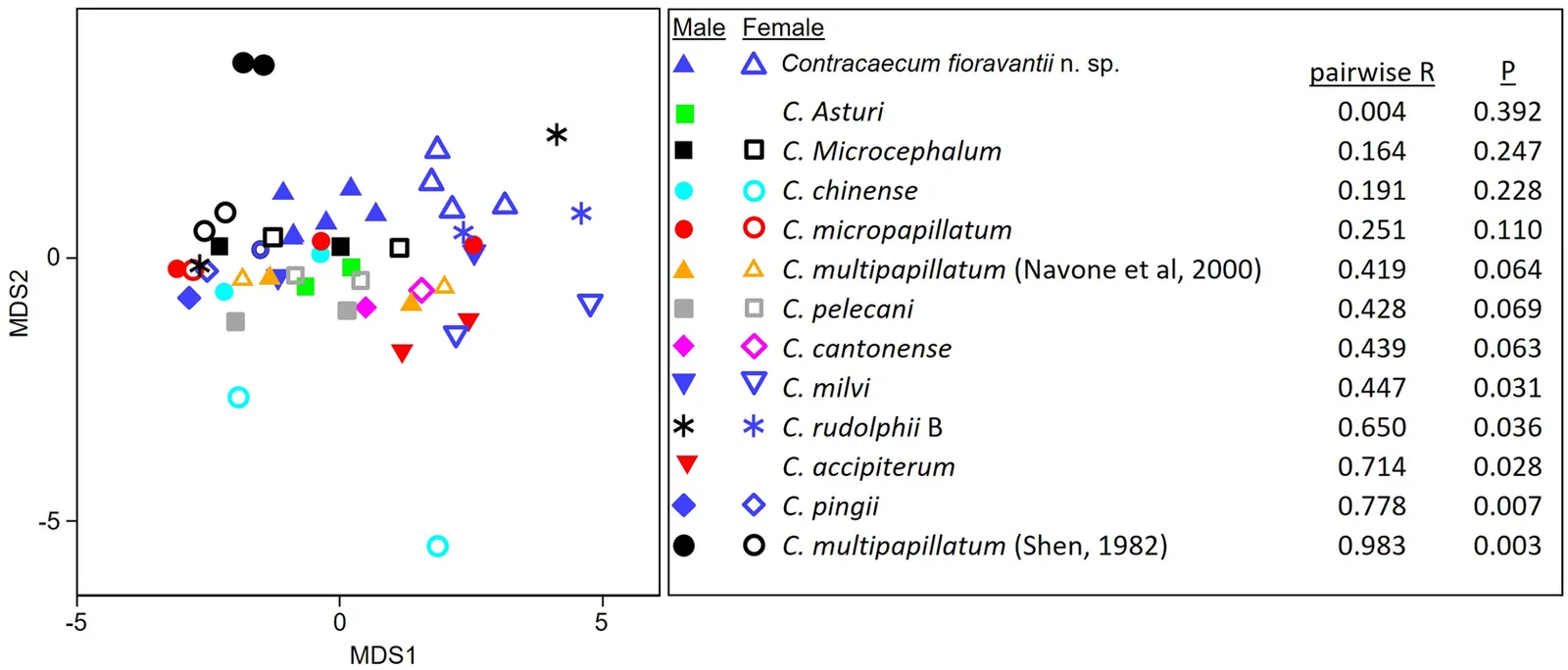
Metric MDS of morphometric resemblance among specimens of *C. fioravantii* n. sp. and data from other species obtained from other studies (1 or more of minimum, maximum, mean). The ordination and ANOSIM tests are based on Euclidean distances calculated from 5 normalized variables (lengths of the whole body, oesophagus, intestinal cecum, ventricular appendix, nerve ring and tail). The 2-dimensional stress of the MDS is 0.13. The results of ANOSIM pairwise tests between *C. fioravantii* n. sp. (2-way crossed with worm sex) are to the right of the ordination, together with MDS symbol legends. Figure 5 long description.

Multivariate analysis revealed varying degrees of morphometric distinction between *C. fioravantii* n. sp. and other species reported or described for the first time in China (Figure 5). In the matrix of Euclidean distances based on 5 morphometric variables, worms differed in terms of species (*R* = 0.345, *P* = 0.0004) and sex (*R* = 0.361, *P* = 0.018). The variables included in the analysis were the lengths of the body, oesophagus, intestinal cecum, ventricular appendix, nerve ring and tail. In pairwise comparisons, *C. fioravantii* n. sp. differed significantly from *C. multipapillatum* Drasche 1882 of Shen (1982), *C. pingii* Xu 1957, *C. accipiterum* Shen 1981, *C. rudolphii* B and *C. milvi* Yamaguti 1935, and with marginal significance from *C. cantonense* Xu 1957, *C. pelecanii* Hu, Lin and Qiu 1997, and *C. multipapillatum* of Navone et al. (2000).

### Molecular results

**Figure 6.**
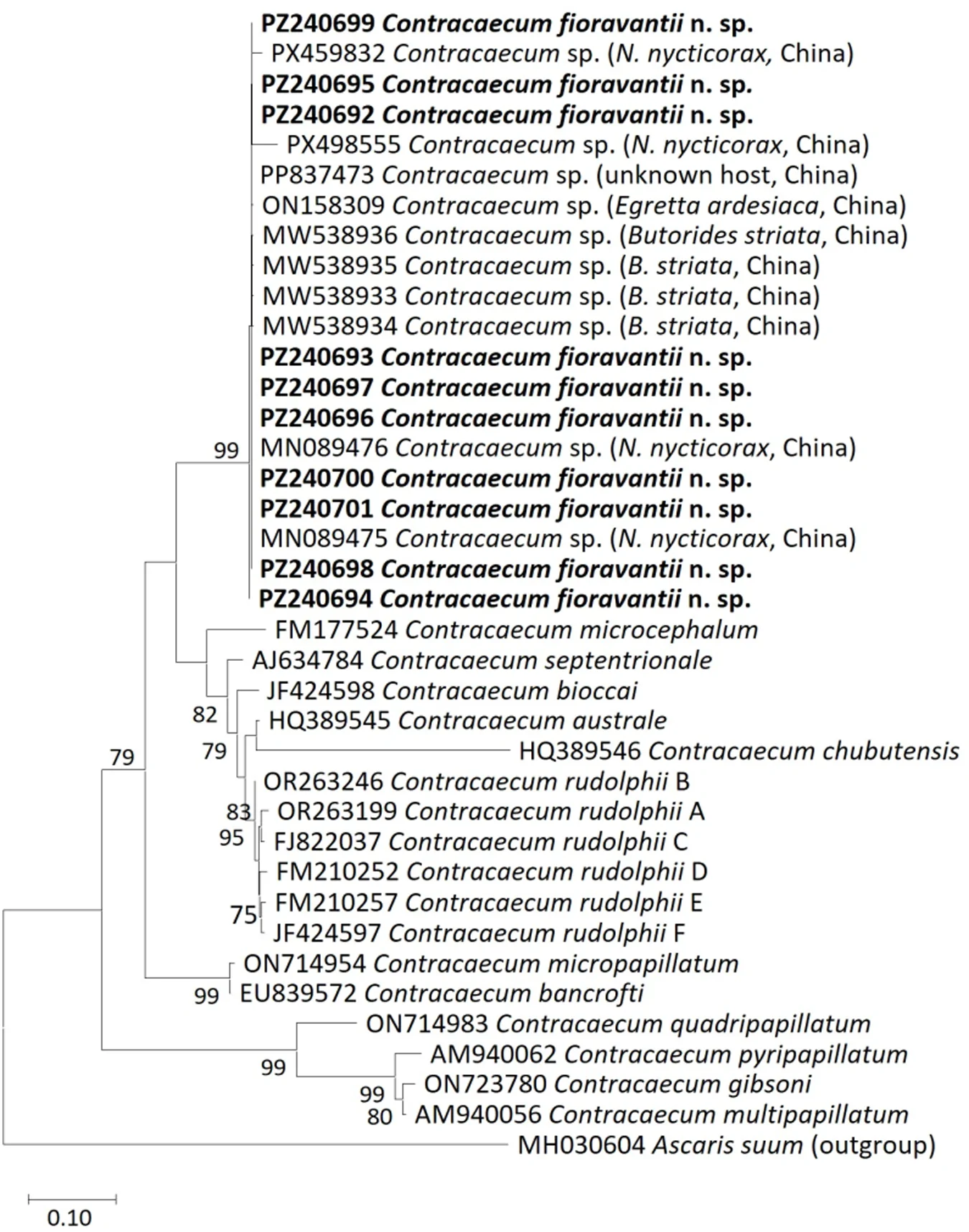
Phylogenetic tree inferred from ITS rDNA sequences showing the position of *C. fioravantii* n. sp. (in bold), reconstructed using the Maximum Likelihood method under the General Time Reversible model of nucleotide substitution. Bootstrap support values (1000 replicates) are shown at the nodes as the percentage of replicate trees in which the associated taxa clustered together. The final dataset comprised 37 nucleotide sequences. Evolutionary analyses were performed in MEGA12 using up to 5 parallel computing threads. Figure 6 long description.

The ITS rDNA of all 10 specimens yielded a product of approximately 850 bp. BLAST searches showed 100% identity with an unidentified *Contracaecum* species collected from *N. nycticorax* in China (MN089475–76, Zhang et al., 2021; PX498555 and PX459832, Yin, 2025, unpublished; PP837473, Long, 2024 unpublished and unknown host), with ON158309 (Deng et al., 2022) from *Egretta ardesiaca* Wagler 1827, and with *Contracaecum* sp. from *Butorides striata* L. in China (MW538933–36, Cheng, 2021, unpublished). The genetic distance (uncorrected *p*) between the new species and the above sequences retrieved from GenBank was 0–0.3%. In phylogenetic analysis, the concatenated ITS1–2 regions of *C. fioravantii* n. sp. formed a well-supported clade (99% bootstrap support) separated from other *Contracaecum* species (Figure 6). This separation clearly supported the morphological differences between our new species.

**Figure 7.**
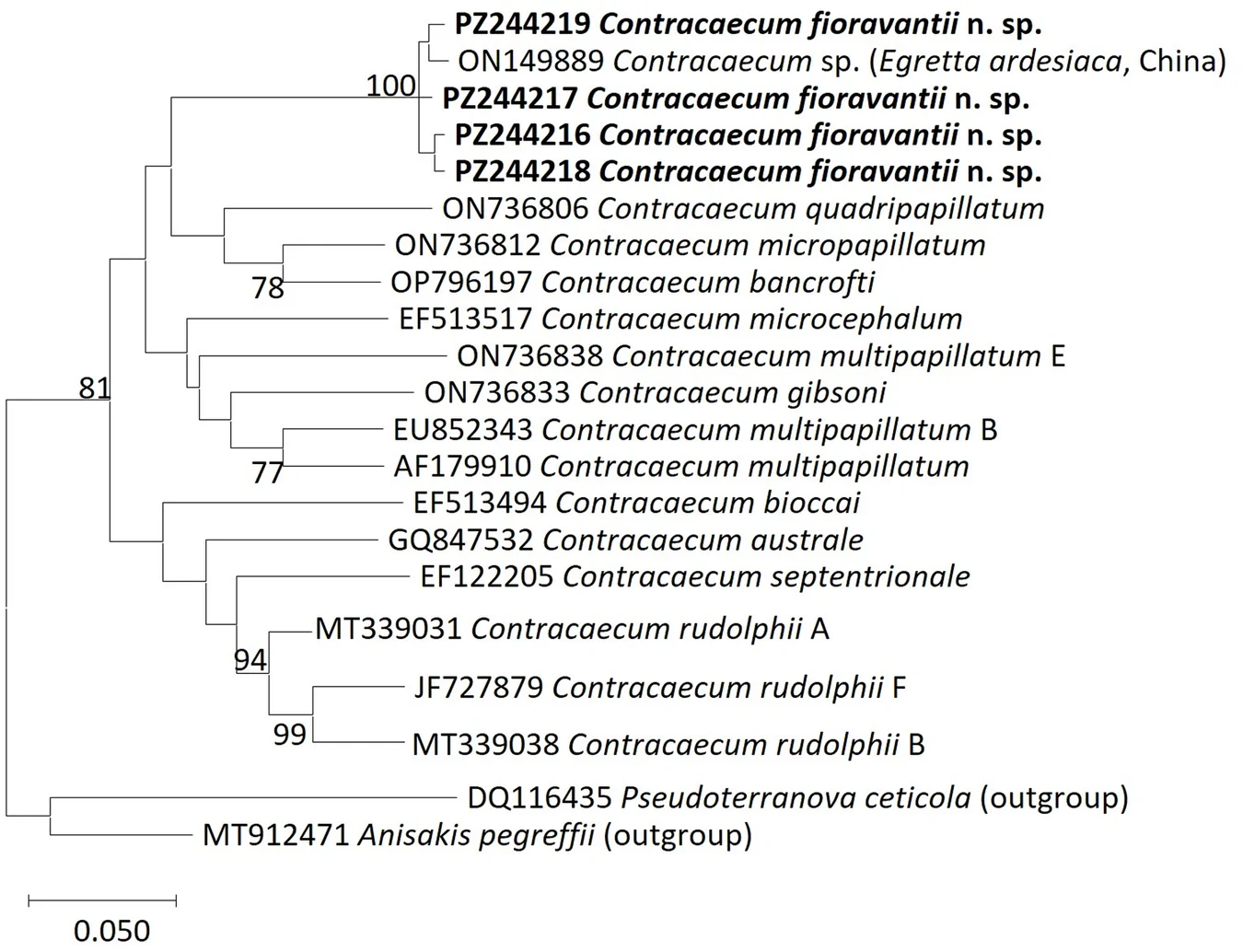
Phylogenetic tree inferred from cox2 mtDNA sequences showing the position of *C. fioravantii* n. sp. (in bold), reconstructed using the Maximum Likelihood method under the Kimura 2-parameter model of nucleotide substitution. Bootstrap support values (1000 replicates) are shown at the nodes as the percentage of replicate trees in which the associated taxa clustered together. The final dataset comprised 23 nucleotide sequences. Evolutionary analyses were performed in MEGA12 using up to 5 parallel computing threads. Figure 7 long description.

Four sequences of *cox2* mtDNA were obtained; BLAST analysis revealed a similarity ranging from 98% to 99.1% with *Contracaecum* sp. (ON149889, Deng et al., 2022) from *E. ardesiaca* in China and an intraspecific *p*-distance of 0.1–0.2%. The ML tree topology clearly places the new species in a well-supported (100% bootstrap) and distinct clade that includes the *Contracaecum* sp. collected in China (Figure 7).

## Discussion

In Israel, *N. nycticorax* is a widespread wetland-associated species occurring in marshes, lakes, rivers and coastal lagoons, where it behaves as both a resident and partial migrant. Its distribution is largely determined by the availability of suitable aquatic habitats, including key areas such as the Hula Reserve and coastal wetlands, despite increasing anthropogenic pressures (Ashkenazi and Yom-tov, 1997; Ashkenazi and Dimentman, 1998; Yom-tov, 2012). The species exhibits an opportunistic diet, feeding on fish, amphibians, crustaceans and aquatic insects, thereby acting as an important host for trophically transmitted parasites, including *Contracaecum* spp.

Interestingly, *C. fioravantii* n. sp. has never been detected in previous molecular surveys of *Contracaecum* in Israel, including extensive sampling of larvae in fish (Davidovich et al., 2022, 2023) and adult *Contracaecum* from other piscivorous birds from the same area as the present study (Caffara et al., 2023a, b). This may indicate that infections in *N. nycticorax* were acquired at other sites along migratory routes, or that the parasite occurs in intermediate hosts not previously investigated.

The identical ITS rDNA sequences and high similarity (99%) in *cox2* mtDNA with unidentified *Contracaecum* specimens from the oesophagus of *N. nycticorax* collected near Beijing, China (Zhang et al., 2021; Deng et al., 2022) strongly suggest that this record corresponds to *C. fioravantii* n. sp. This finding is particularly significant, as it links the new species to the only available complete mitochondrial genome sequences of an unidentified *Contracaecum* species (MN892395, ON149889).

Within the Chinese avifauna, the genus *Contracaecum* is represented by species infecting a wide range of birds, particularly raptors, herons and cormorants (Supplementary Table 1; Zhang et al., 2012; Li et al., 2016). These nematodes have been recorded from various Chinese provinces and have been isolated from a diverse range of hosts (Supplementary Table 1). Some taxa, such as *C. microcephalum, C. multipapillatum* and *C. rudolphii*, exhibit relatively low host specificity, although species boundaries within the *C. multipapillatum* and *C. rudolphii* complexes require further investigation, whereas others appear restricted to a limited number of hosts.

Genetic sequencing (ITS and mitochondrial data) of *C. rudolphii* from cormorants (*Phalacrocorax carbo sinensis*) sampled along the southern coasts of China confirmed their affinity with the species *C. rudolphii* B (Li et al., 2013). However, genetic data are available only for a subset of the *Contracaecum* species from China (e.g. *C. ovale*); nevertheless, many species are readily distinguishable from *C. fioravantii* n. sp. based on morphology. Particularly, *C. ovale* (Linstow 1907) Baylis 1920 (synonym of *C. nehli* and *C. spasskii*; Presswell and Bennett, 2022) is found mainly in grebes and shows a distinct appearance of the anterior end, with markedly rounded upper margins of labia and greatly reduced non-bifurcated interlabia. Moreover, the comparison of our sequences with the ITS1 of *C. ovale* available in GenBank (OL470520, from Australasian crested grebe, *Podiceps cristatus australis* L., Presswell and Bennet, 2022) showed a 1.8% distance with *C. fioravantii* n. sp.

*Contracaecum micropapillatum* and *C. pelecani* show a characteristic pattern of distal papillae with 3 pairs of double papillae. *Contracaecum pingii* has been reported only from the Eurasian goshawk *Astur* (formerly *Accipiter*) *gentilis* L. (Li et al., 2016); however, this species has never been reported after its original description, and has not been recorded in the same host from other parts of the Eurasian continent (Yakovleva and Ieshko, 2025). In China, *A. gentilis* is a reported host for *C. accipiterum*, firstly described by Shen (1981) from Jiangsu province: male individuals of this species are characterized by having 42 pairs of proximal caudal papillae and by 2 pairs of single and 2 pairs of double distal papillae, a pattern distinct from the species under study; also, this species has never been reported after its first description.

*Contracaecum fioravantii* n. sp. shows morphological similarities, with respect to the pattern of distal papillae, with *C. microcephalum* and species within the *C. rudolphii* complex, both already reported from *N. nycticorax*. Nevertheless, the latter 2 species/species complexes markedly differ from *C. fioravantii* n. sp. in the spicule morphometry and morphology of distal posterior end; *C. rudolphii* B also differs in other aspects of overall morphometry from *C. fioravantii* n. sp. (Figure 5). Molecular analyses based on amplification and sequencing of nuclear and mitochondrial markers support this distinction by placing *C. fioravantii* n. sp. in a distinct clade far from *C. multipapillatum* and *C. rudolphii* complexes.

The high similarity with sequences from China suggests that *C. fioravantii* n. sp. may have a broader geographical distribution than currently documented, potentially linked to the migratory behaviour of *N. nycticorax* and other ardeid hosts. This finding also indicates that some previously unidentified *Contracaecum* sequences available in GenBank likely correspond to this species. The recognition of *C. fioravantii* n. sp. underscores the underestimated diversity within *Contracaecum* and the key role of migratory hosts in parasite dispersal. Integrative taxonomy remains essential for resolving species boundaries in this complex genus.

## Supporting information

10.1017/S0031182026102558.sm001 Tedesco et al. supplementary material Tedesco et al. supplementary material

## Data Availability

All the data that support the findings of this study are available from the corresponding author. Molecular sequences are available in GenBank under accession numbers PZ240692-69, PZ240700-01 (ITS rDNA) and PZ244216-19 (*cox2* mtDNA).

## Supplementary material

The supplementary material for this article can be found at https://doi.org/10.1017/S0031182026102558.

## Figure 5 Long description

Two-dimensional metric multidimensional scaling (MDS) ordination illustrating morphometric similarity among specimens of Contracaecum fioravantii n. sp. and comparative data for other Contracaecum species obtained from published studies. The analysis is based on Euclidean distances calculated from six normalized morphometric variables: body length, oesophagus length, intestinal caecum length, ventricular appendix length, nerve ring position, and tail length. Each point represents one specimen or one published morphometric dataset (minimum, maximum, or mean values), with symbols distinguishing species and, for C. fioravantii, male and female specimens. Pairwise ANOSIM test results comparing C. fioravantii with the other taxa are presented alongside the ordination. The two-dimensional MDS solution has a stress value of 0.13, indicating an acceptable representation of the multivariate relationships in two dimensions.

Navigate back to Figure 5.

## Figure 6 Long description

Maximum Likelihood phylogenetic tree inferred from ITS rDNA sequences illustrating the evolutionary relationships of Contracaecum fioravantii n. sp. among other representatives of the genus Contracaecum. The new species is highlighted in bold. Branch labels indicate bootstrap support values based on 1,000 replicates and represent the percentage of replicate trees recovering each clade. The phylogeny was reconstructed under the General Time Reversible model of nucleotide substitution using a dataset of 37 ITS rDNA sequences. Evolutionary analyses were performed in MEGA12 using up to five parallel computing threads.

Navigate back to Figure 6.

## Figure 7 Long description

Maximum Likelihood phylogenetic tree inferred from cox2 mtDNA sequences illustrating the evolutionary relationships of Contracaecum fioravantii n. sp. among other representatives of the genus Contracaecum. The new species is highlighted in bold. Branch labels indicate bootstrap support values based on 1,000 replicates and represent the percentage of replicate trees recovering each clade. The phylogeny was reconstructed under the Kimura 2-parameter model of nucleotide substitution using a dataset of 23 cox2 mtDNA sequences. Evolutionary analyses were performed in MEGA12 using up to five parallel computing threads.

Navigate back to Figure 7.

## References

[ref1] Abollo E, Gestal C and Pascual S (2001) Anisakid infection in the European shag *Phalacrocorax aristotelis aristotelis*. *Journal of Helminthology* 75, 209–214. 10.1079/JOH200051. 11551308

[ref2] Altschul SF, Gish W, Miller W, Myers EW and Lipman DJ (1990) Basic local alignment search tool. *Journal of Molecular Biology* 215, 403–410. 10.1016/S0022-2836(05)80360-2. 2231712

[ref3] Ashkenazi S and Dimentman C (1998) Foraging, roosting, and nesting habitats of the avian fauna of the Agmon wetland, northern Israel. *Wetlands Ecology and Management* 6, 169–187. 10.1023/A:1008488607329.

[ref4] Ashkenazi S and Yom-tov Y (1996) Herons and fish farming in the Huleh Valley, Israel: Conflict or mutual benefit? *Colonial Waterbirds* 19, 143–151. 10.2307/1521958.

[ref5] Ashkenazi S and Yom-tov Y (1997) The breeding biology of the Black-crowned Night-Heron (*Nycticorax nycticorax*) and the Little Egret (*Egretta garzetta*) at the Huleh Nature Reserve, Israel. *Journal of Zoology* 242, 623–641. 10.1111/j.1469-7998.1997.tb05817.x.

[ref6] Barson M and Marshall BE (2004) First record of *Contracaecum* spp. (Nematoda: Anisakidae) in fish-eating birds from Zimbabwe. *Journal of the South African Veterinary Association* 75, 74–78. 10.4102/jsava.v75i2.456. 15456162

[ref7] Baruš V, Sergeeva TP, Sonin MD, Ryzhikov KM and Ryšavý B (1978) Systematic Part. In Ryšavý B and Ryzhikov KM (eds.), *Helminths of Fish-Eating Birds of the Palaearctic Region. Helminths of Fish Eating Birds, Vol 1*. Dordrecht: Springer 71–93. 10.1007/978-94-009-9972-5_3.

[ref8] Caffara M, Tedesco P, Davidovich N, Locke SA, Gustinelli A, King R, Nuytten M, Nuzzo M and Fioravanti ML (2023a) Advancing understanding of the taxonomy and diversity of the genus *Contracaecum* in the great white pelican (*Pelecanus onocrotalus*). *Parasitology Research* 122, 315–331. 10.1007/s00436-022-07732-z. 36434318

[ref9] Caffara M, Tedesco P, Davidovich N, Rubini S, Luci V, Cantori A, Glogowski PA, Fioravanti ML and Gustinelli A (2023b) Molecular and morphological studies on *Contracaecum rudolphii* A and *C. rudolphii* B in great cormorants (*Phalacrocorax carbo sinensis*) from Italy and Israel. *Parasitology* 150, 1040–1051. 10.1017/S0031182023000902. PMC10941213 37859396

[ref10] Cram EB (1927) Bird parasites of the nematode suborders Strongylata, Ascaridata, and Spirurata. *Bulletin of the United States National Museum* 140, 1–465. 10.5479/si.03629236.140.1.

[ref11] Davidovich N, Tedesco P, Caffara M, Luci V, Cantori A, Morick D, Fioravanti ML and Gustinelli A (2023) Low prevalence of *Contracaecum* third-stage larvae parasitizing sea of Galilee fisheries: 1-year survey after 57 years of no information. *Food and Waterborne Parasitology* 32, e00204. 10.1016/j.fawpar.2023.e00204. PMC10372363 37520837

[ref12] Davidovich N, Tedesco P, Caffara M, Yasur-landau D, Gustinelli A, Drabkin V, Minkova E, Aflalo O, Morick D and Fioravanti ML (2022) Morphological description and molecular characterization of *Contracaecum* larvae (Nematoda: Anisakidae) parasitizing market-size hybrid tilapia (*Oreochromis aureus* x *Oreochromis niloticus*) and red drum (*Sciaenops ocellatus*) farmed in Israel. *Food and Waterborne Parasitology* 26, e00147. 10.1016/j.fawpar.2022.e00147. PMC8857656 35243050

[ref13] Deng YP, Li R, Wang HM, Liu GH and Tu Y (2022) Complete mitochondrial genome of *Contracaecum* sp. (Nematoda: Ascarididae) from Night Herons in China. *Journal of Nematology* 54, 20220048. 10.2478/jofnem-2022-0048. PMC9676673 36457371

[ref14] Dies JI, Ramón N and Prosper J (2003) Winter breeding by Black-Crowned Night Heron in Eastern Spain. *Waterbirds* 26, 379–382. 10.1675/1524-4695(2003)026[0379:WBBBNH]2.0.CO;2.

[ref15] Dziekońska-rynko J, Mierzejewska K and Hliwa P (2015) Parasitic helminths in grey heron (*Ardea cinerea*) chicks. *Biologia* 70, 279–282. 10.1515/biolog-2015-0022.

[ref16] Endo N and Sawara Y and in Japanese with English summary (2000) Diel rhythmic activity and foraging site utilization of the Black-crowned Night Heron (*Nycticorax nycticorax*) in its breeding season. *Japan Journal of Ornithology* 48, 183–196. 10.3838/jjo.48.183.

[ref17] Fagerholm HP (1991) Systematic implications of male caudal morphology in ascaridoid nematode parasites. *Systematic Parasitology* 19, 215–228.

[ref18] Groves JA, Achatz TJ, Kinsella JM, Calhoun DM, Wilcox JT, Greiman SI and Johnson PTJ (2025) A Survey of endohelminth diversity of birds collected in the San Francisco Bay Area. *Journal of Parasitology* 111, 658–668. 10.1645/24-123. 41087023

[ref19] Hall TA (1999) BioEdit: A user-friendly biological sequence alignment editor and analysis program for Windows 95/98/NT. *Nucleic Acids Symposium Series* 41, 95–98.

[ref20] Hartwich G (1964) Revision der vogelparasitischen nematoden mitteleuropas II. Die Gattung *Contracaecum* Railliet and Henry, 1912 (Ascaridoidea). *Mitteilungen Aus Dem Zoologischen Museum in Berlin* 40, 15–53.

[ref21] IUCN. 2025. The IUCN Red List of Threatened Species. Version 2025-2. Available at: www.iucnredlist.org. (accessed 2 April 2026).

[ref22] Kumar S, Stecher G, Suleski M, Sanderford M, Sharma S and Tamura K (2024) MEGA12: Molecular evolutionary genetic analysis version 12 for adaptive and green computing. *Molecular Biology and Evolution* 41, msae263. 10.1093/molbev/msae263. PMC11683415 39708372

[ref23] Li L, Gibson DI and Zhang LP (2016) An annotated catalogue of the ascaridoid nematode parasites of Chinese vertebrates. *Systematic Parasitology* 93, 1–35. 10.1007/s11230-015-9617-5. 26739284

[ref24] Li L, Xu Z and Zhang LP (2013) Further studies on *Contracaecum spasskii* Mozgovoi, 1950 and *C. rudolphii* Hartwich, 1964 (sensu lato) (Ascaridida: Anisakidae) from piscivorous birds in China. *Systematic Parasitology* 84, 225–236. 10.1007/s11230-012-9399-y. 23404759

[ref25] Mattiucci S, Paoletti M, Webb SC, Sardella N, Timi JT, Berland B and Nascetti G (2008) Genetic relationships among species of *Contracaecum* Railliet & Henry, 1912 and *Phocascaris* Höst, 1932 (Nematoda: Anisakidae) from pinnipeds inferred from mitochondrial cox2 sequences, and congruence with allozyme data. *Parasite* 15, 408–419. 10.1051/parasite/2008153408. 18814715

[ref26] Mattiucci S, Sbaraglia GL, Palomba M, Filippi S, Paoletti M, Cipriani P and Nascetti G (2020) Genetic identification and insights into the ecology of *Contracaecum rudolphii* A and *C. rudolphii* B (Nematoda: Anisakidae) from cormorants and fish of aquatic ecosystems of Central Italy. *Parasitology Research* 119, 1243–1257. 10.1007/s00436-020-06658-8. 32239289

[ref27] Mhaisen FT and Abdul-ameer K (2021) Checklist of fish hosts of species of *Contracaecum* Railliet & Henry, 1912 (Nematoda: Ascaridida: Anisakidae) in Iraq. *Biological and Applied Environmental Research* 5, 281–306. 10.51304/baer.2021.5.2.281.

[ref28] Navarro P, Lluch J and Font E (2005) The component helminth community in six sympatric species of Ardeidae. *Journal of Parasitology* 91, 775–779. 10.1645/GE-3342.1. 17089743

[ref29] Navone GT, Etchegoin JA and Cremonte F (2000) *Contracaecum multipapillatum* (Nematoda: Anisakidae) from *Egretta alba* (Aves: Ardeidae) and comments on other species of this genus in Argentina. *Journal of Parasitology* 86, 807–810. 10.1645/0022-3395(2000)086[0807:CMNAFE]2.0.CO;2 10958460

[ref30] Pérez JJ, De Lope F, Turégano B and De la Cruz C (1991) The food of black-crowned night-heron nestlings in extremadura (W Spain). *Ardeola* 38, 277–287.

[ref31] Presswell B and Bennett J (2022) Gastrointestinal helminth parasites of the threatened Australasian crested grebe (*Podiceps cristatus australis*, Gould 1844) in New Zealand, with descriptions of *Baruscapillaria kamanae* n. sp. (Nematoda: Trichuridae) and *Cryptocotyle. Micromorpha n. sp.* (Trematoda: Opisthorchiidae). *Systematic Parasitology* 99, 217–239. 10.1007/s11230-022-10022-y. PMC8885541 35124752

[ref32] Quiroga MA, León EJ, Beltzer AH and Olguín PF (2013) Diet of Black-crowned Night-Herons (Nycticorax nycticorax) in a wetland of the Paraná River’s alluvial valley. *Ekoloji* 22(88), 43–50. 10.5053/ekoloji.2013.886.

[ref33] Santoro M, D’alessio N, Di Prisco F, Veneziano V, Galiero G, Cerrone A, Barca L, Kinsella JM and Aznar FJ (2026) Helminth communities of herons (Aves: Ardeidae) in southern Italy. *Parasitology International* 65, 340–346. 10.1016/j.parint.2016.04.002. 27091547

[ref34] Shamsi S and Butcher AR (2011) First report of human anisakidosis in Australia. *Medical Journal of Australia* 194, 199–200. 10.5694/j.1326-5377.2011.tb03772.x. 21401462

[ref35] Shen SX (1981) On new species of nematodes parasitic in birds from Taihu region, Jiangsu Province, China. *Acta Zootaxonomica Sinica* 6, 359–364. In Chinese, English summary.

[ref36] Shen SX (1982) First report of *Contracaecum multipapillatum* from China. *Acta Zootaxonomica Sinica* 6, 359–364. In Chinese.

[ref37] Sitko J and Heneberg P (2015) Composition, structure and pattern of helminth assemblages associated with central European herons (Ardeidae). *Parasitology International* 64, 100–112. 10.1016/j.parint.2014.10.009. 25449288

[ref38] Syrota Y, Lisitsyna O, Greben O, Kornyushin V and Du Preez L (2021) The helminth infracommunities in three species of herons (Aves: Ardeidae) from Ukraine. *Parasitology International* 85, 102442. 10.1016/j.parint.2021.102442. 34454105

[ref39] Yakovleva G and Ieshko E (2025) The helminth fauna of the Eurasian goshawk (*Accipiter gentilis*) in northwest Russia. *Raptor Journal* 19, 29–37. 10.2478/srj-2025-0004.

[ref40] Yom-tov Y (2012) Israel’s breeding avifauna: A century of dramatic change. *Biological Conservation* 147, 16–26. 10.1016/j.biocon.2012.01.003.

[ref41] Yoshida K, Tsuji M and Iwata K (2012) Molecular identification and prevalence of *Contracaecum* nematodes in *Nycticorax nycticorax* in Japan. *Parasitology Research* 110, 1115–1123. 10.1007/s00436-011-2717-8.

[ref42] Zhang Q, Meng M, Huang C, Feng S, Liu J, Tang Y, Fan Y, Yuan G, Han S, Luo J, Zhao B and He H (2021) Identification and genetic characterization of *Contracaecum* sp. (Nematoda: Anisakidae) from China. *Integrative Zoology* 16, 929–938. 10.1111/1749-4877.12465. 32652769

[ref43] Zhang S, Bu Y, Huang G, Wen Q and Zhang L (2012) A checklist of parasitic nematodes (Nematoda) from birds (Aves) in China. *Zootaxa* 3446, 1–31. 10.11646/zootaxa.3446.1.1.

[ref44] Zhu X, Gasser RB, Podolska M and Chilton NB (1998) Characterisation of anisakid nematodes with zoonotic potential by nuclear ribosomal DNA Sequences. *International Journal for Parasitology* 28, 1911–1921. 10.1016/S0020-7519(98)00150-7. 9925272

